# Supplementary material for: Corporate financial distress prediction with multiperiod annual report data: A fusion deep neural network model
Source: PLoS One. 2025 Sep 26;20(9):e0333064. doi: 10.1371/journal.pone.0333064 (PMC12469393; doi:10.1371/journal.pone.0333064)
Supplement: Attachment — Submitted filename: Response to Reviewers.docx [file pone.0333064.s001.docx]

Dear Editors:

This is a resubmitted manuscript by Chongren Wang, Pimei Gong, Jiawang Li and Zhiyi Wang, entitled “Corporate financial distress prediction with multiperiod annual report data: A fusion deep neural network model” with Manuscript [PONE-D-25-01170]. We have carefully revised this paper according to the editorial decision letter one by one and explains how the paper has been changed point-to-point. See Section 1 for a detailed description.

The authors have read and approved this version of the article, and due care has been taken to ensure the integrity of the work. No part of this paper has been published or submitted elsewhere. No conflict of interest exits in the submission of this manuscript.

This paper elaborately details a fused deep neural network model based on multiperiod annual report text and financial indicator data to predict corporate financial distress. The main contributions of this study are as follows:

(1) Development of a feature fusion model to extract semantic features from long texts.

This study applies multiperiod financial report texts to the field of financial distress prediction and designs a novel neural network architecture tailored to the characteristics of multiperiod financial report texts. This approach enhances the prediction performance of financial distress models that incorporate long texts from multiperiod financial reports. First, the pretrained Longformer model is utilized to compute word vectors for the long texts in financial reports. Subsequently, the global features of the text were extracted via bidirectional long short-term memory (Bi-LSTM), and the local features were extracted via a Text Convolutional Neural Network (TextCNN). A feature-level fusion method is then used to construct multiperiod semantic features of long texts, offering a new perspective for extracting semantics from multiperiod financial report texts.

(2) A dynamic financial distress prediction model is constructed on the basis of multiperiod annual report data.

A dynamic financial distress prediction model is constructed on the basis of multimodal data from multiperiod annual report texts and financial indicators. First, multiple fully connected neural networks are used to process multiperiod financial indicator data. Next, a fusion model is designed to handle multiperiod annual report texts by extracting long texts from the Management Discussion and Analysis (MD&A) sections of multiple years. The feature fusion model is then used to compute the global and local semantic features of these long texts, and the results are integrated as multiperiod text semantic features. Finally, a neural network combines the extracted multiperiod textual semantics and multiperiod financial features for predictive computation.

We believe the paper may be of particular interest to the readers of your journal. Correspondence should be addressed to Prof. Gong at the following address, phone, and email address:

School of Management Science and Engineering

Shandong University of Finance and Economics,

Erhuandong Road,

Lixia District,

Jinan 250014, P.R. China.

Tel: (+86) 19861818113

E-mail: *m1926017049@gmail.com*

Thanks very much for your attention to my paper. We are looking forward to hearing from you soon.

Sincerely yours,

Pimei Gong

**Detailed Response to Reviewers**

Manuscript ID: [PONE-D-25-01170]

Paper Title: Corporate financial distress prediction with multiperiod annual report data: A fusion deep neural network model

Dear Editors and Reviewers,

Thanks for your letter and for the reviewers’ constructive comments concerning our manuscript entitled “Corporate financial distress prediction with multiperiod annual report data: A fusion deep neural network model” (Manuscript [PONE-D-25-01170]). These comments are all valuable and very helpful for improving our paper, as well as the important guiding significance to our research. We have modified our paper according to these comments carefully. We have used the "Track Changes" function in Microsoft Word to revise the manuscript without making any unmarked modifications. Please see purple colored parts for the revisions. We deeply hope that our revised paper could meet your requirements.

**Section 1: a) and b)**

***a. The entire comments made by Reviewers***

**Reviewer #1**:

The paper proposes a Fusion Deep Neural Network model based on Multiple annual Reports Text data and Financial data (MTF-FDNN). This model is capable of extracting long-text features from multi-period corporate annual reports as well as features from multi-period financial indicators. To further improve the manuscript, the following suggestions are given:

1、In the paper, some figures in the manuscript are a little blurry, please improve the clarity.

2、The formatting specifications throughout the manuscript need to be carefully checked and revised. For example, formulas need to be centered.

3、Since there are some papers in this topic, the contributions of the manuscript should be better summarized and listed.

4、While the introduction sets the context, a more explicit literature review section could better situate the study within the broader research landscape, such as Financial distress prediction with annual reports-based deep textual feature extraction: A hybrid approach, EVADE Targeted Adversarial False Data Injection Attacks for State Estimation in Smart Grid, and so on. These references could provide valuable insights into your research.

5、Add a section on the limitations of the work and future work in this paper.

**Reviewer #2**

The introduction section

1. The introduction could be strengthened by incorporating more recent literature to better contextualize the study. For example, the sentence: “Previous studies have shown that predictive models that combine textual and financial data can significantly increase the accuracy of financial distress predictions.” lacks citations for the referenced studies. Similarly, the authors state: “In the early stages of financial distress prediction research, scholars domestically and internationally relied primarily on financial indicators from a single fiscal year, applying machine learning or deep learning techniques for prediction. In recent years, text mining has become a hot topic in academia.” Again, no supporting references are provided. Including relevant citations would enhance the credibility of these claims.

2. A clearer transition from the problem statement to the proposed solution would improve the overall flow of the introduction.

3. The choice of Longformer, Bi-LSTM, and TextCNN requires stronger theoretical justification. Why is Longformer better than other transformers? Why were Bi-LSTM and TextCNN combined instead of alternative architectures (e.g., hierarchical attention networks)? Providing a rationale for these choices would strengthen the methodological foundation of the study.

4. Adding a brief description of the structure of the article at the end of the introduction would enhance its clarity and help guide the reader through the following sections.

The related works section

5. Some of the references cited in the manuscript are relatively old (e.g., Beaver, 1966). Additionally, the manuscript does not contain many sources, and most references are older than five years.

For example, you state “Hajek P. et al. [15] applied XGBoost and AdaBoost algorithms for classification analysis of samples that integrate textual metrics. García V. et al. [16] further improved the classification accuracy of models by combining ensemble strategies such as bagging and random subspace methods including more recent studies would provide a more current context.”, the mentioned authors, as well as others, have more recent studies in this subject area. Including newer literature would help provide a more up-to-date context.

6. The organization of related studies into subsections is appreciated, as it enhances the clarity of the article. However, the discussion of individual studies is too brief. The authors introduce the problems each study addresses but do not provide an in-depth discussion of the methodologies or results. A more detailed analysis of prior work, including quantitative performance metrics (e.g., accuracy rates of previous models), would improve the quality of this section. Furthermore, while the limitations of prior studies are mentioned, the research gaps they leave unaddressed are not explicitly stated. Identifying these gaps more clearly would help to better understand the described studies within The related works section.

The experiments and results section

7. The CSMAR dataset is used, but there is no justification for why this dataset is representative. More information about the dataset, such as the number of companies, the time span covered, and other relevant characteristics, should be provided. Additionally, there is no discussion of cross-validation or alternative training/test splits to ensure the model avoids overfitting. The authors should explain why they chose a 60:20:20 split and whether they experimented with other ratios (e.g., 70:20:10).

8. The comparison between traditional machine learning models and MTF-FDNN is clear, but the claims of superiority would be more robust with statistical significance testing (e.g., p-values). Adding such analyses would strengthen the validity of the results.

Conclusion and future works sections

9. The conclusion could be more reflective, discussing the broader impact and potential applications of the proposed model.

10. A deeper comparison between the proposed MTF-FDNN model and previous financial distress prediction models mentioned in the Related works section is missing. While the article presents results, it does not explicitly compare them to previous state-of-the-art models in financial distress prediction. The authors should clarify why MTF-FDNN is superior to BERT-based models, CNN-only models, or other hybrid architectures.

11. A discussion of computational efficiency and trade-offs between accuracy and interpretability would be beneficial. Addressing these aspects would provide a more balanced assessment of the model's practical implications.

12. There is no discussion including the limitations and implications of the research.

***b. The Point-to-Point responses to the Reviewers.***

**Reply to Editors**

**We are grateful to you for providing us with the opportunity to strengthen the paper. The detailed explanations can be seen below.**

**Reviewer #1**:

**Comment 1:** Please ensure that your manuscript meets PLOS ONE's style requirements, including those for file naming.

**Response:** We have revised the manuscript accordingly to ensure compliance with the journal’s guidelines, including formatting, file naming, and citation styles.

**Comment 2:** Please note that PLOS ONE has specific guidelines on code sharing for submissions in which author-generated code underpins the findings in the manuscript. In these cases, we expect all author-generated code to be made available without restrictions upon publication of the work.

**Response:** Additional explanation on code sharing was provided in the data availability section, and necessary code is available on GitHub (https://github.com/1926017049/MTF-FDNN-model).

**Comment 3:** Please state what role the funders took in the study. If the funders had no role, please state: "The funders had no role in study design, data collection and analysis, decision to publish, or preparation of the manuscript."

**Response:** The funders had no role in study design, data collection and analysis, decision to publish, or preparation of the manuscript and declaration has been added to the funding section.

**Comment 4:** PLOS requires an ORCID iD for the corresponding author in Editorial Manager on papers submitted after December 6th, 2016. Please ensure that you have an ORCID iD and that it is validated in Editorial Manager. To do this, go to ‘Update my Information’ (in the upper left-hand corner of the main menu), and click on the Fetch/Validate link next to the ORCID field. This will take you to the ORCID site and allow you to create a new iD or authenticate a pre-existing iD in Editorial Manager.

**Response:** We have ensured that the corresponding author has a registered and validated ORCID iD in Editorial Manager.

**Thanks again for your kind reminder. We deeply hope that our revised paper could meet your requirements. Thank you very much again for your kind help.**

**Reply to Reviewer #1**

**We are grateful to you for identifying the weaknesses in our paper and providing us with the opportunity to strengthen the paper. The detailed explanations can be seen below.**

**Reviewer #1**: The paper proposes a Fusion Deep Neural Network model based on Multiple annual Reports Text data and Financial data (MTF-FDNN). This model is capable of extracting long-text features from multi-period corporate annual reports as well as features from multi-period financial indicators.

**Comment 1:** In the paper, some figures in the manuscript are a little blurry, please improve the clarity.

**Response:** We have addressed the issue of blurry figures in the manuscript by replacing the affected images with high-resolution versions. Additionally, all figures were carefully checked and adjusted for sharpness to ensure consistency throughout the document.

**Comment 2:** The formatting specifications throughout the manuscript need to be carefully checked and revised. For example, formulas need to be centered.

**Response:** We have thoroughly revised the manuscript formatting according to the journal's guidelines, with specific attention to the issues you raised:

Formula Alignment: All formulas have been centered and consistently numbered.

**Comment 3:** Since there are some papers in this topic, the contributions of the manuscript should be better summarized and listed.

**Response:** In the introduction section, we have clearly summarized the main contributions of our manuscript. Specifically, our contributions include:

(1) Development of a feature fusion model to extract semantic features from long texts.

This study applies multiperiod financial report texts to the field of financial distress prediction and designs a novel neural network architecture tailored to the characteristics of multiperiod financial report texts. This approach enhances the prediction performance of financial distress models that incorporate long texts from multiperiod financial reports. First, the pretrained Longformer model is utilized to compute word vectors for the long texts in financial reports. Subsequently, the global features of the text were extracted via bidirectional long short-term memory (Bi-LSTM), and the local features were extracted via a Text Convolutional Neural Network (TextCNN). A feature-level fusion method is then used to construct multiperiod semantic features of long texts, offering a new perspective for extracting semantics from multiperiod financial report texts.

(2) A dynamic financial distress prediction model is constructed on the basis of multiperiod annual report data.

A dynamic financial distress prediction model is constructed on the basis of multimodal data from multiperiod annual report texts and financial indicators. First, multiple fully connected neural networks are used to process multiperiod financial indicator data. Next, a fusion model is designed to handle multiperiod annual report texts by extracting long texts from the Management Discussion and Analysis (MD&A) sections of multiple years. The feature fusion model is then used to compute the global and local semantic features of these long texts, and the results are integrated as multiperiod text semantic features. Finally, a neural network combines the extracted multiperiod textual semantics and multiperiod financial features for predictive computation.

To further clarify our contributions, we have refined the wording in the introduction to ensure that readers can better understand the novelty and significance of our work. We hope this revision addresses the reviewer's concern. Thank you for your insightful suggestion.

**Comment 4:** While the introduction sets the context, a more explicit literature review section could better situate the study within the broader research landscape, such as financial distress prediction with annual reports-based deep textual feature extraction: A hybrid approach, EVADE Targeted Adversarial False Data Injection Attacks for State Estimation in Smart Grid, and so on. These references could provide valuable insights into your research.

**Response:** We sincerely appreciate your insightful suggestion. To better situate our study within the broader research landscape, we have incorporated more recent literature in the introduction, which helps establish a clearer research context, including references such as *Financial Distress Prediction with Annual Reports-Based Deep Textual Feature Extraction: A Hybrid Approach* , among others. Additionally, we have revised the literature review section to provide a more comprehensive discussion of related works.

**Comment 5:** Add a section on the limitations of the work and future work in this paper.

**Response:** Thanks for your reminder. In response, we have added a dedicated section discussing the limitations of our study and potential directions for future research. This section provides a critical reflection on the constraints of our approach and outlines possible improvements and extensions, which we believe will enhance the clarity and completeness of our work.

**Thanks again for your kind reminder. We deeply hope that our revised paper could meet your requirements. Thank you very much again for your kind help.**

**Reply to Reviewer #2**

**We are grateful to you for identifying the weaknesses in our paper and providing us with the opportunity to strengthen the paper. The detailed explanations can be seen below.**

**Reviewer #2**:

**The introduction section**

**Comment 1:** The introduction could be strengthened by incorporating more recent literature to better contextualize the study. For example, the sentence: “Previous studies have shown that predictive models that combine textual and financial data can significantly increase the accuracy of financial distress predictions.” lacks citations for the referenced studies. Similarly, the authors state: “In the early stages of financial distress prediction research, scholars domestically and internationally relied primarily on financial indicators from a single fiscal year, applying machine learning or deep learning techniques for prediction. In recent years, text mining has become a hot topic in academia.” Again, no supporting references are provided. Including relevant citations would enhance the credibility of these claims.

**Response:** In response to this concern, we have revised the introduction by incorporating more recent literature to better contextualize our study. Specifically, we have added relevant citations to support the claims regarding the effectiveness of predictive models, as well as the historical development of financial distress prediction research. These additions enhance the credibility and robustness of our discussion.

**Comment 2:** A clearer transition from the problem statement to the proposed solution would improve the overall flow of the introduction.

**Response:** To enhance the logical flow of the introduction, we have refined the transition from the problem statement to the proposed solution. Specifically, we have clarified the research gap, emphasized the limitations of existing approaches, and provided a smoother lead-in to our proposed method. These revisions improve the coherence and readability of the introduction.

**Comment 3:** The choice of Longformer, Bi-LSTM, and TextCNN requires stronger theoretical justification. Why is Longformer better than other transformers? Why were Bi-LSTM and TextCNN combined instead of alternative architectures (e.g., hierarchical attention networks)? Providing a rationale for these choices would strengthen the methodological foundation of the study.

**Response:** We have added a stronger theoretical justification for the choice of Longformer, Bi-LSTM, and TextCNN in our introduction section. Specifically, we have elaborated on the advantages of Longformer, which employs a sliding window and global attention mechanism to effectively capture long-range dependencies in lengthy financial reports while maintaining lower computational complexity.

Additionally, we have clarified the rationale for combining Bi-LSTM and TextCNN. Bi-LSTM is well-suited for financial report analysis as it captures bidirectional contextual dependencies, which are essential for understanding text with strong sequential relationships. Meanwhile, TextCNN extracts local features through convolutional filters, effectively capturing phrase-level information. The combination of Bi-LSTM and TextCNN allows our model to leverage both global and local textual features, enhancing the representation of financial reports.

Furthermore, we plan to introduce a hierarchical attention mechanism (e.g., Hierarchical Attention Networks) in future work to construct a three-level semantic representation at the word, sentence, and document levels. This will further enhance long-text contextual modeling capabilities and improve the effectiveness of financial text analysis.

**Comment 4:** Adding a brief description of the structure of the article at the end of the introduction would enhance its clarity and help guide the reader through the following sections.

**Response:** We have added a brief description of the article’s structure at the end of the introduction. This addition helps improve the clarity of our manuscript and provides readers with a clear roadmap of the subsequent sections. Specifically, we now outline the key contents of each section to guide the reader through the study. We appreciate the reviewer’s valuable feedback, which has helped enhance the organization and readability of our manuscript.

**The related works section**

**Comment 5:** Some of the references cited in the manuscript are relatively old (e.g., Beaver, 1966). Additionally, the manuscript does not contain many sources, and most references are older than five years.

For example, you state “Hajek P. et al. [15] applied XGBoost and AdaBoost algorithms for classification analysis of samples that integrate textual metrics. García V. et al. [16] further improved the classification accuracy of models by combining ensemble strategies such as bagging and random subspace methods including more recent studies would provide a more current context. ..”, the mentioned authors, as well as others, have more recent studies in this subject area. Including newer literature would help provide a more up-to-date context.

**Response:** We have thoroughly revised the literature review section, incorporating a substantial number of more recent references to ensure the manuscript reflects the latest advancements in financial distress prediction. While classic studies such as Beaver (1966) provide historical context, we have supplemented them with recent research published within the last five years to strengthen the foundation of our study. Specifically, we have included recent studies on deep learning-based financial distress prediction, advanced text mining techniques for financial data, and the integration of multimodal data sources. These additions provide a more comprehensive and up-to-date review of the field.

**Comment 6:** The organization of related studies into subsections is appreciated, as it enhances the clarity of the article. However, the discussion of individual studies is too brief. The authors introduce the problems each study addresses but do not provide an in-depth discussion of the methodologies or results. A more detailed analysis of prior work, including quantitative performance metrics (e.g., accuracy rates of previous models), would improve the quality of this section. Furthermore, while the limitations of prior studies are mentioned, the research gaps they leave unaddressed are not explicitly stated. Identifying these gaps more clearly would help to better understand the described studies within the related works section.

**Response:** We revised the Related Work section to include a more detailed analysis of previous studies. For each subsection, we expand the discussion to explore the methodology, outcomes, and performance metrics in more depth, such as the accuracy of the model in question. In addition, we provide a clear overview of the limitations of previous studies and the research gaps they have not addressed in each subsection.

**The experiments and results section**

**Comment 7:** The CSMAR dataset is used, but there is no justification for why this dataset is representative. More information about the dataset, such as the number of companies, the time span covered, and other relevant characteristics, should be provided. Additionally, there is no discussion of cross-validation or alternative training/test splits to ensure the model avoids overfitting. The authors should explain why they chose a 60:20:20 split and whether they experimented with other ratios (e.g., 70:20:10).

**Response:** In the Experiments and Results section, we have clarified that the CSMAR dataset is widely used in the field of economic and financial research and is highly representative. It covers a broad range of financial and economic data, including financial statements of listed companies, market transaction data, and corporate governance structures, making it a comprehensive resource for financial distress prediction.

To enhance the robustness of our results, we conducted five-fold cross-validation and reported the average performance to ensure the reliability of our findings. Additionally, we experimented with different data splitting ratios, including 70:20:10 and 60:20:20, and performed multiple rounds of training and evaluation to analyze their impact on model performance.

**Comment 8:** The comparison between traditional machine learning models and MTF-FDNN is clear, but the claims of superiority would be more robust with statistical significance testing (e.g., p-values). Adding such analyses would strengthen the validity of the results.

**Response:** We have added statistical significance testing to our comparison between traditional machine learning models and MTF-FDNN. Specifically, we performed significance tests (e.g., p-values) to assess the robustness of the observed performance improvements. These analyses confirm that the differences in performance between the models are statistically significant, further strengthening the validity of our results. We believe these additions enhance the credibility of our findings and provide a more rigorous comparison of our model with traditional approaches.

**Conclusion and future works sections**

**Comment 9:** The conclusion could be more reflective, discussing the broader impact and potential applications of the proposed model.

**Response:** We have expanded the conclusion section to include a deeper discussion of the broader impact and potential applications of our proposed model. Specifically, we explore how the model can be applied to different industries and financial contexts, as well as its potential for enhancing predictive accuracy in financial distress prediction across various datasets. Additionally, we discuss the implications of our findings for future research and practical implementation in both academic and real-world settings. We believe these additions help convey the wider relevance of our work and provide a clearer vision of its impact.

**Comment 10:** A deeper comparison between the proposed MTF-FDNN model and previous financial distress prediction models mentioned in the Related works section is missing. While the article presents results, it does not explicitly compare them to previous state-of-the-art models in financial distress prediction. The authors should clarify why MTF-FDNN is superior to BERT-based models, CNN-only models, or other hybrid architectures.

**Response:** We have added a more in-depth comparison between the MTF-FDNN model and previous state-of-the-art financial distress prediction models mentioned in the related work section. Specifically, we have clarified why MTF-FDNN outperforms models based on BERT, pure CNN models, and other hybrid architectures. We explain that the combination of multiple features from both textual and financial data in MTF-FDNN allows for a more comprehensive representation of the data, enabling it to capture long-range dependencies and contextual information more effectively than BERT-based models alone. Additionally, unlike purely CNN-based models that focus on local feature extraction, MTF-FDNN combines the strengths of both Bi-LSTM and TextCNN, addressing both global and local feature extraction, which is crucial for financial report analysis.

We believe these additions strengthen the comparison section and provide a clearer justification for the advantages of MTF-FDNN over existing models.

**Comment 11:** A discussion of computational efficiency and trade-offs between accuracy and interpretability would be beneficial. Addressing these aspects would provide a more balanced assessment of the model's practical implications.

**Response:** We added a brief discussion on the trade-offs between computational efficiency, and accuracy. We explained that MTF-FDNN improves computational efficiency by using Longformer, which reduces the complexity of processing long texts. Additionally, MTF-FDNN enhances prediction accuracy by combining multi-period financial data and text data from annual reports, outperforming traditional machine learning models. We believe these additions provide a more balanced evaluation of the model's practical implications.

**Comment 12:** There is no discussion including the limitations and implications of the research.

**Response:** We have added a discussion of the limitations and implications of our research. We acknowledge that further optimization of hyperparameters is possible, and the model could benefit from incorporating more diverse data sources beyond annual report texts. Additionally, we address the class imbalance issue and suggest that future work could explore techniques like data augmentation or GANs to improve performance. We believe these additions provide a more complete understanding of the study’s limitations and future directions.

**Thanks again for your kind reminder. We deeply hope that our revised paper could meet your requirements. Thank you very much again for your kind help.**
