# Supplementary material for: Corporate financial distress prediction with multiperiod annual report data: A fusion deep neural network model
Source: PLoS One. 2025 Sep 26;20(9):e0333064. doi: 10.1371/journal.pone.0333064 (PMC12469393; doi:10.1371/journal.pone.0333064)
Supplement: Attachment — Submitted filename: Response_to_Reviewers_auresp_2.docx [file pone.0333064.s002.docx]

Dear Editors:

This is a resubmitted manuscript by Chongren Wang, Pimei Gong, Jiawang Li and Zhiyi Wang, entitled “Corporate financial distress prediction with multiperiod annual report data: A fusion deep neural network model” with Manuscript [PONE-D-25-01170] - [EMID:b7df50fc92330332]. We have carefully revised this paper according to the editorial decision letter one by one and explains how the paper has been changed point-to-point. See Section 1 for a detailed description.

The authors have read and approved this version of the article, and due care has been taken to ensure the integrity of the work. No part of this paper has been published or submitted elsewhere. No conflict of interest exits in the submission of this manuscript.

This paper elaborately details a fused deep neural network model based on multiperiod annual report text and financial indicator data to predict corporate financial distress. The main contributions of this study are as follows:

(1) Development of a feature fusion model to extract semantic features from long texts.

This study applies multiperiod financial report texts to the field of financial distress prediction and designs a novel neural network architecture tailored to the characteristics of multiperiod financial report texts. This approach enhances the prediction performance of financial distress models that incorporate long texts from multiperiod financial reports. First, the pretrained Longformer model is utilized to compute word vectors for the long texts in financial reports. Subsequently, the global features of the text were extracted via bidirectional long short-term memory (Bi-LSTM), and the local features were extracted via a Text Convolutional Neural Network (TextCNN). A feature-level fusion method is then used to construct multiperiod semantic features of long texts, offering a new perspective for extracting semantics from multiperiod financial report texts.

(2) A dynamic financial distress prediction model is constructed on the basis of multiperiod annual report data.

A dynamic financial distress prediction model is constructed on the basis of multimodal data from multiperiod annual report texts and financial indicators. First, multiple fully connected neural networks are used to process multiperiod financial indicator data. Next, a fusion model is designed to handle multiperiod annual report texts by extracting long texts from the Management Discussion and Analysis (MD&A) sections of multiple years. The feature fusion model is then used to compute the global and local semantic features of these long texts, and the results are integrated as multiperiod text semantic features. Finally, a neural network combines the extracted multiperiod textual semantics and multiperiod financial features for predictive computation.

We believe the paper may be of particular interest to the readers of your journal. Correspondence should be addressed to Prof. Gong at the following address, phone, and email address:

School of Management Science and Engineering

Shandong University of Finance and Economics,

Erhuandong Road,

Lixia District,

Jinan 250014, P.R. China.

Tel: (+86) 19861818113

E-mail: *m1926017049@gmail.com*

Thanks very much for your attention to my paper. We are looking forward to hearing from you soon.

Sincerely yours,

Pimei Gong

**Detailed Response to Reviewers**

Manuscript ID: [PONE-D-25-01170] - [EMID:b7df50fc92330332]

Paper Title: Corporate financial distress prediction with multiperiod annual report data: A fusion deep neural network model

Dear Editors and Reviewers,

Thanks for your letter and for the reviewers’ constructive comments concerning our manuscript entitled “Corporate financial distress prediction with multiperiod annual report data: A fusion deep neural network model” (Manuscript [PONE-D-25-01170] - [EMID:b7df50fc92330332]). These comments are all valuable and very helpful for improving our paper, as well as the important guiding significance to our research. We have modified our paper according to these comments carefully. We have used the "Track Changes" function in Microsoft Word to revise the manuscript without making any unmarked modifications. Please see purple colored parts for the revisions. We deeply hope that our revised paper could meet your requirements.

**Section 1: a) and b)**

***a. The entire comments made by Reviewers***

**Reviewer #1:** Please revise carefully based on the comments raised. Although experimental results are provided, the paper would benefit from comparative analysis with current state-of-the-art approaches. More in depth comparison and analysis should be given in the manuscript.

**Reviewer #2:** The authors deal with an interesting topic. The topic is well work out at the required level in term of content and of formal aspect.

***b. The Point-to-Point responses to the Reviewers.***

**Reply to Reviewer #1**

**We are grateful to you for providing us with the opportunity to strengthen the paper. The detailed explanations can be seen below.**

**Reviewer #1**:

Please revise carefully based on the comments raised. Although experimental results are provided, the paper would benefit from comparative analysis with current state-of-the-art approaches. More in depth comparison and analysis should be given in the manuscript.

**Response:** Thank you for your valuable comments. We have added a new section, 4.4 "Parameter Sensitivity Analysis," in the manuscript, where we conduct a detailed analysis of several key parameters to further enrich the experimental results and discussion. Please refer to the main text for details. We hope these additions better demonstrate the advantages and performance of our method.
